# Supplementary material for: LRRK2 is a negative regulator of Mycobacterium tuberculosis phagosome maturation in macrophages
Source: EMBO J. 2018 May 22;37(12):e98694. doi: 10.15252/embj.201798694 (PMC6003659; doi:10.15252/embj.201798694)
Supplement: Supplementary file 1 — Appendix [file EMBJ-37-e98694-s001.pdf]

Appendix for

LRRK2 is a negative regulator of *Mycobacterium tuberculosis* phagosome maturation in macrophages

Härtlova, Herbst et al.,

Contents:

Appendix Materials and Methods

LDH assay

Cells were seeded in a 96 well plate and infected with Mtb at a MOI of 1 to 5. For the determination of LDH release, supernatants were collected at 72 h post infection and LDH release was measured using the CytoTox 96 Non-Radioactive Cytotoxicity Assay from Promega according to the manufacturer's protocol. Maximum LDH released was determined by adding Triton-X-100 to a final concentration of 2 % v/v for 45 min before collecting the supernatants.

Image analysis

Bacterial replication: measurement of bacterial replication in single cells was performed as described before (Lerner et al., 2016). Briefly, cells were seeded on cover slips, infected with Mtb-GFP at a MOI=1 and fixed every 24 h over a time course of 72 h. Analysis was performed using FIJI (US National Institutes of Health, USA). Leica SP5 files (.lif) were opened using the BioFormats plugin of FIJI. Custom written macros were used to analyse the images as follows: Measuring intracellular bacterial growth (measuring the bacterial GFP signal per cell): A region of interest (ROI) (i.e. a macrophage containing Mtb-GFP (green channel)) was drawn around using the line tool within FIJI, using bright field as a guide. The ROI was duplicated, and anything outside the ROI was cleared. Then, the images were split into their constitutive colour channels, and the green channel (corresponding to the GFP signal from the bacteria) was subject to a threshold. The bacteria were selected and the RAWIntDen measurement was

taken (this is the sum of all the pixels originating from the GFP signal, which correlates with the number of intracellular bacteria).

Lysosomal morphology: Lysosomal morphology was analysed using FIJI (US National Institutes of Health, USA). In order to determine the average size of lysosomes per cells and the number of lysosomes per cell, ROIs were drawn around single cells using the bright field image as a guide. The whole image was thresholded on the channel of the lysosomal marker and lysosomal morphology parameters were calculated for each individual ROI.

Marker association to phagosomes: dynamic association of 2xFYVE-GFP or Rubicon-GFP to red-fluorescent BSA-coated latex beads (2 µm carboxylated FluoSpheres, ThermoFisher Scientific) was performed as described before (Pei, Repnik et al., 2014). Briefly, RAW264.7 macrophages were plated at $1x10^5$ on WillCo-dish® glass-bottom dishes and transfected with 1 ug plasmid DNA using Lipofectamine 3000 (ThermoFisher Scientific) one day prior to the experiment. Cells were then incubated with the beads and imaging performed using a Leica TCS SP5 II microscope (Leica Microsystems) equipped with AOBS, a HC PLAOP CS2 63.0x1.40 OIL objective and an environmental control chamber providing 37°C, 5 %CO_2 and 20-30% humidity. Images were acquired in 15 to 32 sec intervals over a time of 2 h and analysed in FIJI (US National Institutes of Health, USA) using a macro described previously (Schnettger, Rodgers et al., 2017). In brief, association was measured by automated analysis of the mean relative fluorescence intensity in a 2-pixel wide ring around the beads. Since transfection levels vary between cells, mean fluorescent intensities were normalised to the maximum intensity observed for each individual uptake event.

Phagocytosis assay

Phagocytosis of beads was evaluated by incubating 3 µm carboxylated- (COOH) or amino- (NH2) Alexa Flour 488 BSA-coated silica beads (Kisker-Biotech) at 1:1000 and 1:300 dilutions with BMDMs in 96-well plates for 30 min at 37°C. Beads were replaced with 100 µl trypan blue to quench the fluorescence of non-internalised particles. After aspirating trypan blue, the fluorescence was

measured in a SpectraMax Gemini EM Fluorescence Microplate Reader, set at excitation/emission wavelengths 495/519 nm.

References

Pei G, Repnik U, Griffiths G, Gutierrez MG (2014) Identification of an immune-regulated phagosomal Rab cascade in macrophages. J Cell Sci 127: 2071-82

Schnettger L, Rodgers A, Repnik U, Lai RP, Pei G, Verdoes M, Wilkinson RJ, Young DB, Gutierrez MG (2017) A Rab20-Dependent Membrane Trafficking Pathway Controls M. tuberculosis Replication by Regulating Phagosome Spaciousness and Integrity. Cell host & microbe 21: 619-628 e5
