# Supplementary material for: LRRK2 is a negative regulator of Mycobacterium tuberculosis phagosome maturation in macrophages
Source: EMBO J. 2018 May 22;37(12):e98694. doi: 10.15252/embj.201798694 (PMC6003659; doi:10.15252/embj.201798694)
Supplement: Supplementary file 2 — Expanded View Figures PDF [file EMBJ-37-e98694-s002.pdf]

Expanded View Figures

Figure EV1. Loss of LRRK2 activity targets Mtb to phagolysosomes and limits Mtb replication.

A Confirmation of efficient LRRK2 kinase inhibition by GSK2578215A 24 h after infection of BMDM or iPSDM with Mtb. Representative image of whole cell lysate Western blotted for LRRK2 pS935, total LRRK2 and α-tubulin.

B, C LDH assay performed at 72 h post-infection as toxicity control for CFU experiments in Fig 1. Data show mean ± SD from three technical replicates. LDH assays were routinely performed for CFU and ELISA experiments.

D CFUs in RAW264.7 cells pre-treated with GSK2578215A (1 μM) or DMSO control for 2 h and infected with Mtb-eGFP (MOI = 1). *$P < 0.5$, ***$P < 0.001$ by Student's *t*-test corrected for multiple comparison. Data show mean ± SD. One out of two experiments shown.

E RAW264.7 cells were pre-treated with 1 μM GSK2578215A or DMSO (Control) for 2 h, and LAMP-1 recruitment at 24 h post-infection was assessed using confocal microscopy.

F Quantification of panel (E). Data show mean ± SEM from three independent experiments. **$P < 0.01$ by Student's *t*-test.

G Mtb growth analysed by single cell imaging in WT, LRRK2 KO and LRRK2 G2019S KI BMDM. Data show mean ± SEM from three independent experiments. ***$P < 0.001$ by Student's *t*-test corrected for multiple comparisons.

H Mtb growth analysed by single cell imaging in WT, LRRK2 G2019S KI BMDM and LRRK2 G2019S KI BMDM treated with GSK2578215A for 2 h. Data show mean ± SEM from three independent experiments. **$P < 0.001$ by Student's *t*-test corrected for multiple comparisons.

I CFU in WT BMDM, WT BMDM treated with GSK2578215A and LRRK2 KO BMDM left untreated or pre-activated with IFN-γ (100 U/ml over night). Data show mean ± SD from technical replicates. One representative experiment out of three experiments shown. ***$P < 0.001$ by Student's *t*-test corrected for multiple comparison.

© 2018 The Francis Crick Institute

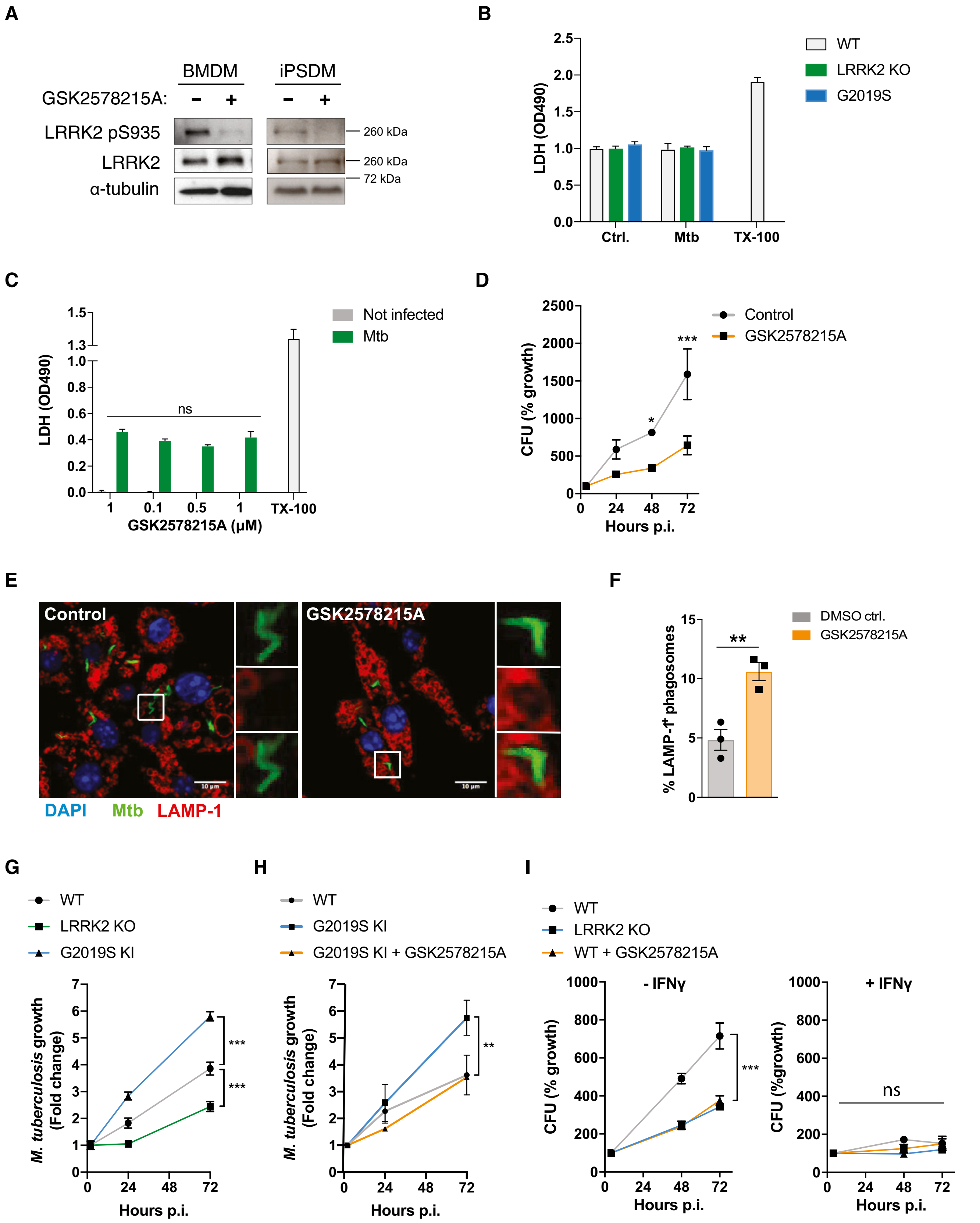
A
BMDM
iPSDM
GSK2578215A:
LRRK2 pS935
LRRK2
α-tubulin
260 kDa
260 kDa
72 kDa
B
WT
LRRK2 KO
G2019S
LDH (OD490)
Ctrl.
Mtb
TX-100
C
Not infected
Mtb
LDH (OD490)
ns
GSK2578215A (µM)
TX-100
D
Control
GSK2578215A
CFU (% growth)
Hours p.i.
E
Control
GSK2578215A
DAPI
Mtb
LAMP-1
F
DMSO ctrl.
GSK2578215A
% LAMP-1+ phagosomes
G
WT
LRRK2 KO
G2019S KI
M. tuberculosis growth (Fold change)
Hours p.i.
H
WT
G2019S KI
G2019S KI + GSK2578215A
M. tuberculosis growth (Fold change)
Hours p.i.
I
WT
LRRK2 KO
WT + GSK2578215A
- IFNγ
+ IFNγ
CFU (% growth)
CFU (%growth)
ns
Hours p.i.

Figure EV1.

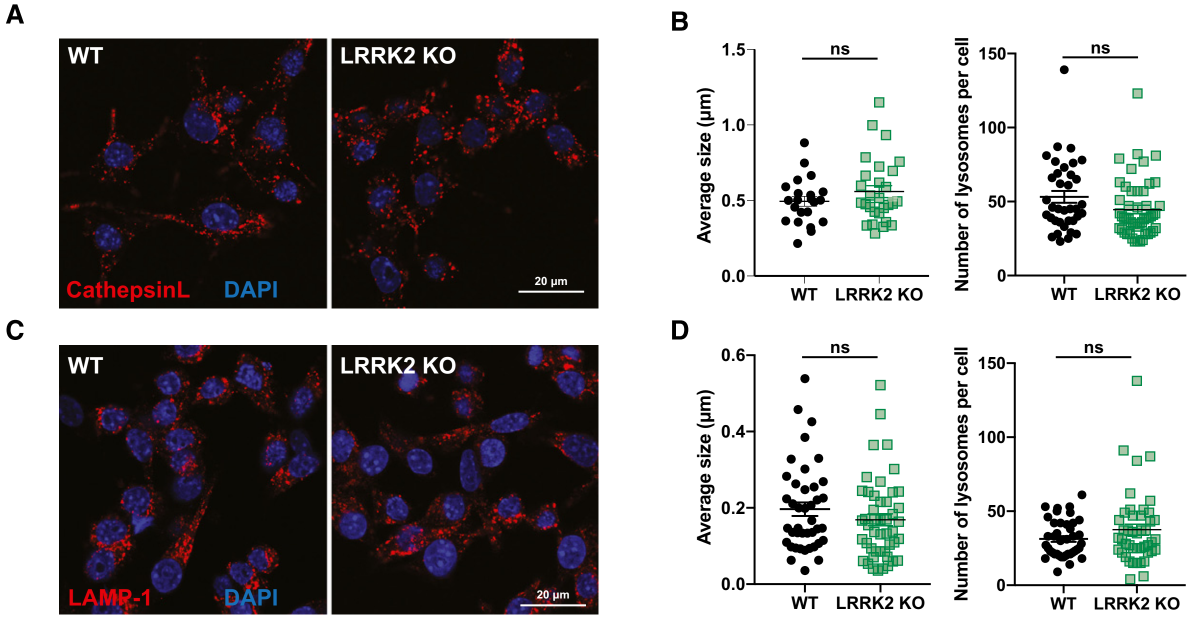

Figure EV2. LRRK2 KO has no major impact on lysosomal morphology.

A Representative images of WT and LRRK2 KO BMDM incubated with cathepsin L probe for 30 min.
B Quantification of (A), showing average lysosomal size and numbers. Each dot represents a single cell.
C Representative images of WT and LRRK2 KO BMDMs stained for LAMP-1.
D Quantification of (C), showing average lysosomal size and numbers. Each dot represents a single cell.

Data information: Data show mean ± SEM from three independent experiments. ns = not significant by Student's *t*-test.

 © 2018 The Francis Crick Institute

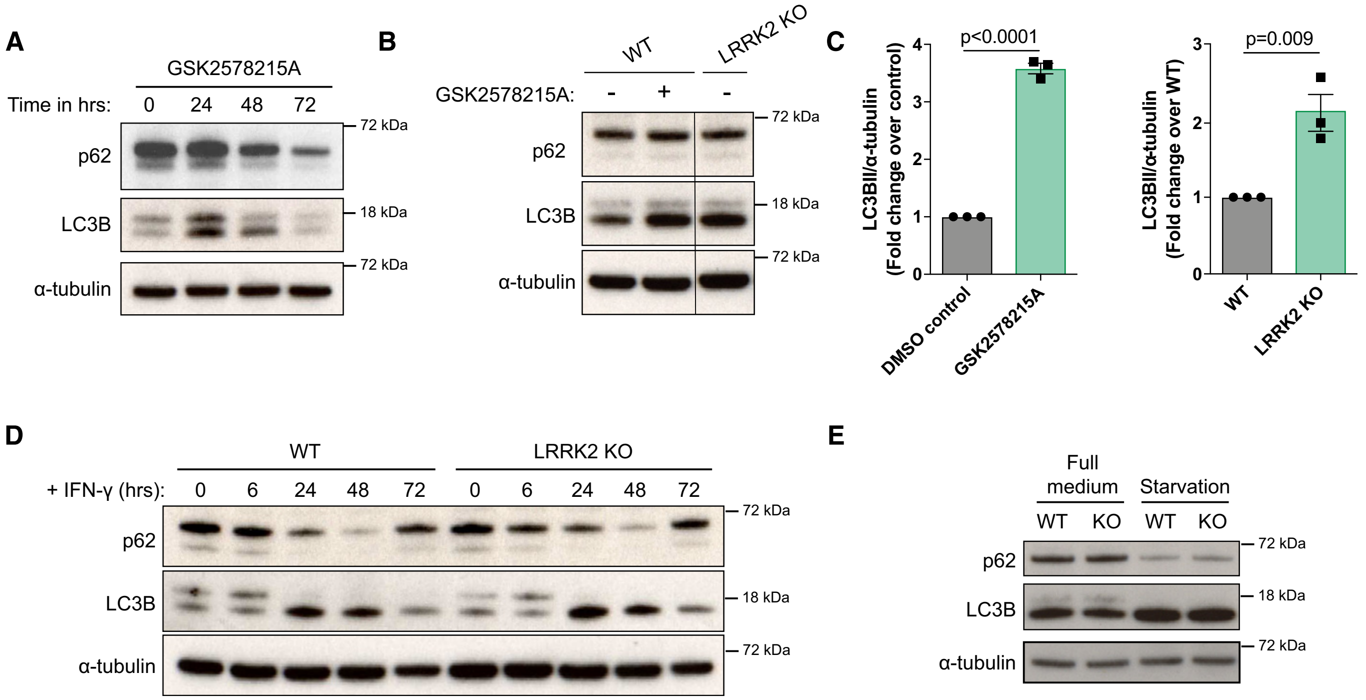

Figure EV3. Role of LRRK2 in autophagy regulation.

A WT BMDMs were treated with 1 μM GSK2578215A, and p62 and LC3B levels were monitored by Western blotting over time. α-tubulin was used as a loading control.
B WT and LRRK2 KO BMDMs were treated or not with 1 μM GSK2578215A and p62 and LC3B levels were monitored by Western blotting at 24 h after treatment. α-Tubulin was used as a loading control.
C Quantification of (B). Each dot represents an independent experiment. Data show mean + SEM analysed by Student's *t*-test.
D WT and LRRK2 KO BMDMs were treated with 100 U/ml IFN-γ, and p62 and LC3B levels were monitored by Western blotting over time. α-Tubulin was used as a loading control.
E WT and LRRK2 KO BMDMs were left in full medium or starved in HBSS for 4 h, and p62 and LC3B levels were monitored by Western blotting. α-Tubulin was used as a loading control.

© 2018 The Francis Crick Institute

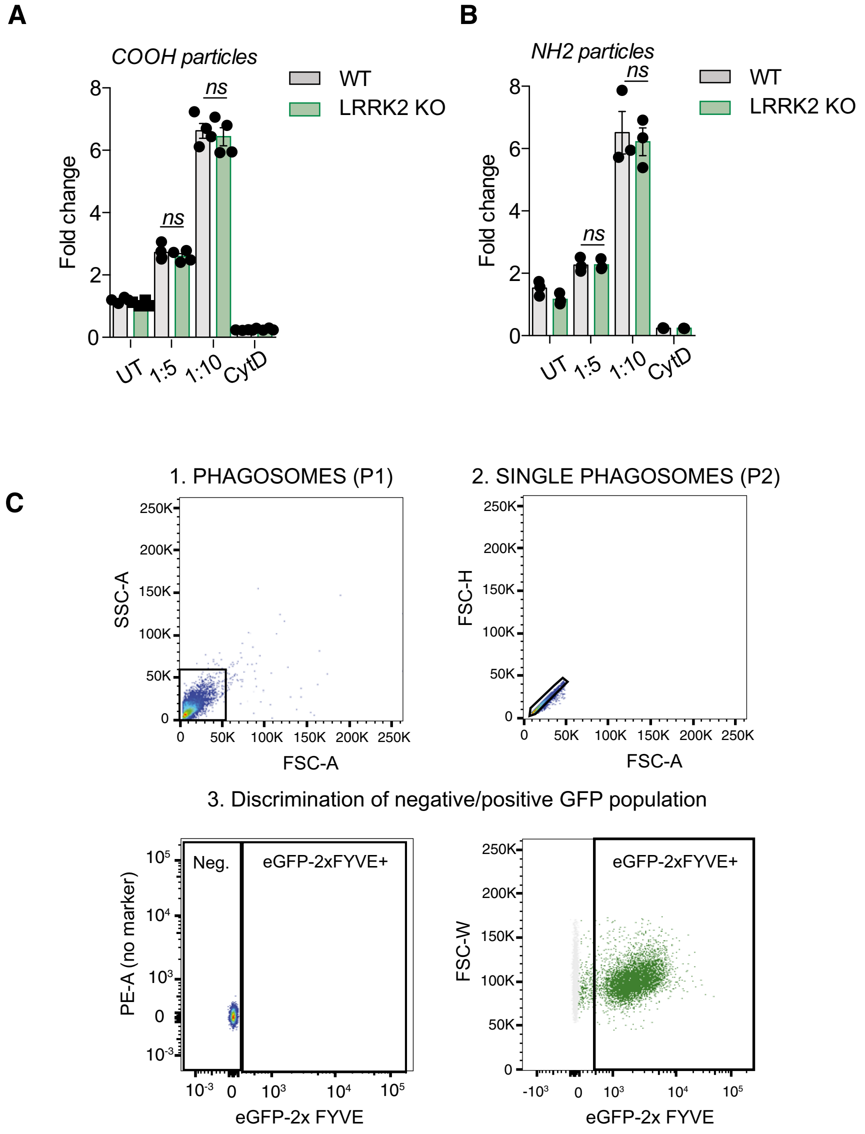

Figure EV4. Loss of LRRK2 does not regulate phagocytic uptake of COOH and NH_2 beads in macrophages.

A, B Measurement of the rate of phagocytosis of AF488-coated carboxylated A and amino B beads in WT and LRRK2 KO macrophages. Beads were added to macrophages in 1:300 and 1:1,000 ratio for 30 min, cytochalasin D (1 μM, 1 h pre-treatment) was used as the inhibitor of phagocytosis. Data show mean ± SEM of three biological replicates. ns: non-significant. Student's *t*-test.

C Gating strategy for phagoFACS and measurement of 2xFYVE-domain-GFP-positive phagosomes. Panel 1 shows beads alone, and panel 2 shows phagosomes containing beads.

© 2018 The Francis Crick Institute

A

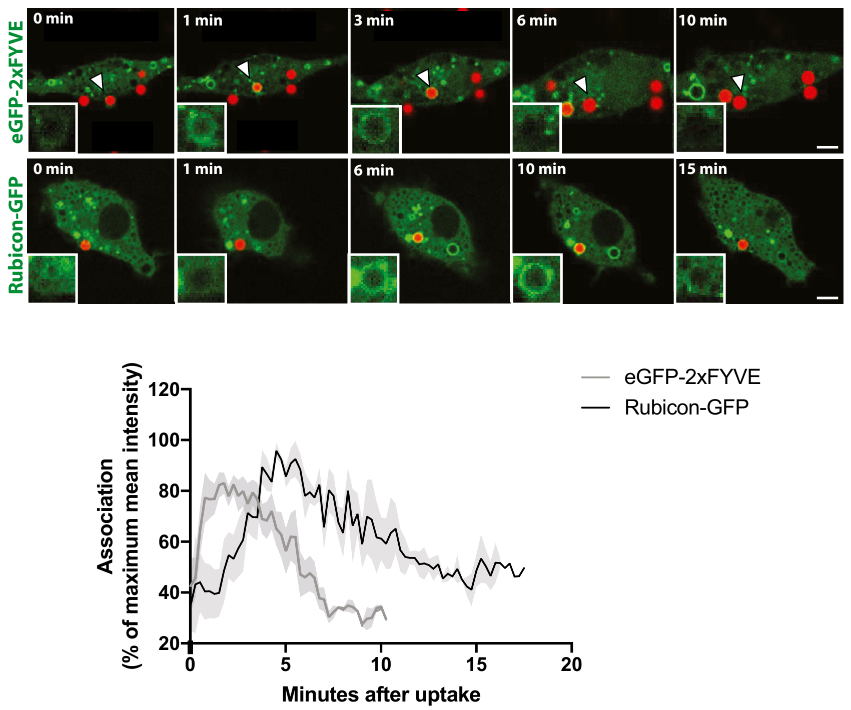

B

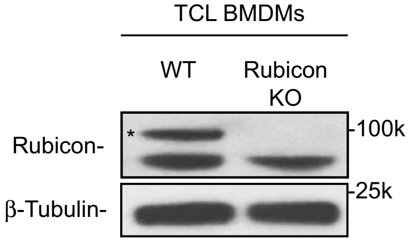

Figure EV5. Rubicon recruitment follows PI(3)P recruitment to phagosome.

A RAW264.7 macrophages were transfected with either 2xFYVE-domain-GFP or Rubicon-GFP plasmid and incubated with 2 μm BSA-coated red-fluorescent carboxylated latex beads. Images were taken every 15 s, and mean fluorescent intensities around the beads were analysed using ImageJ. Graph shows % of maximum mean fluorescence ± SEM, n = 10 phagosomes. The arrowhead marks the bead shown in the zoomed panels. Scale bars = 5 μm.

B Control demonstrating specificity of the anti-Rubicon antibody for Western blotting. Total cell lysates of WT and Rubicon KO BMDMs were blotted for Rubicon and β-tubulin.

© 2018 The Francis Crick Institute
